# Lithium in Older Adults: A Community Services Audit and Service Development Project Focusing on Blood Monitoring, Clinic Assesments and GP Correspondence

**DOI:** 10.1192/bjo.2026.11679

**Published:** 2026-06-30

**Authors:** Huda Khan, Odile Hally

**Affiliations:** 1 National Forensic Mental Health Services, Dublin, Ireland; 2 North Dublin Mental Health Services, Dublin, Ireland

## Abstract

**Aims:**

Lithium is an effective mood stabilizer for older adults but carries increased risks due to age-related physiological changes, comorbidities, and reduced renal function. Regular monitoring of lithium levels, renal and thyroid function, and electrolytes is essential to prevent toxicity, ensure safe dosing, and optimize treatment outcomes.

**Methods:**

This audit aimed to evaluate lithium monitoring practices within psychiatry of old age services in Mental Health Services for Older Persons. It focused on the monitoring of lithium levels and relevant blood tests, as well as the completion of comprehensive lithium assessments to ensure safe and effective use in older adults keeping in view of the vulnerability of this age group.

**Results:**

A retrospective review of all lithium patients attending the service was conducted, assessing lithium evaluations, documentation, monitoring of side effects, and adherence to recommended blood monitoring standards following NICE and Maudesley guidelines.

In Cycle 1, deficiencies were noted in documenting side effects, medication interactions, and regular monitoring of eGFR and physical health. In response, Cycle 2 introduced a service development project with structured monitoring tools. Lithium flowsheets were implemented to record blood tests every three months, and lithium stickers were used by nursing staff to document results in clinical notes. A two-page clinical assessment form, incorporating tick-boxes for side effects, interactions, and patient education, ensured all areas were systematically addressed. A GP template was also developed to prompt timely blood tests.

These interventions resulted in significant improvements in lithium monitoring and documentation. Additionally, a formal lithium policy was established within the North Dublin Mental Health Services for Older People Community Mental Health Team, supporting consistent and safe practice.

**Conclusion:**

Overall, the implementation of structured monitoring tools and policy measures enhanced adherence to recommended blood tests, improved documentation of side effects, and strengthened patient education. These initiatives have collectively improved the quality, safety, and effectiveness of lithium prescribing in older adults.

